# The variant at *TGFBRAP1* is significantly associated with type 2 diabetes mellitus and affects diabetes‐related miRNA expression

**DOI:** 10.1111/jcmm.13885

**Published:** 2018-11-20

**Authors:** Song Yang, Xiaotian Chen, Mengyao Yang, Xianghai Zhao, Yanchun Chen, Hailong Zhao, Chunlan Liu, Chong Shen

**Affiliations:** ^1^ Department of Cardiology Affiliated Yixing People's Hospital of Jiangsu University People's Hospital of Yixing City Yixing China; ^2^ Department of Epidemiology School of Public Health Nanjing Medical University Nanjing China; ^3^ Department of Clinical Epidemiology Geriatric Hospital of Nanjing Medical University Jiangsu Province Geriatric Institute Nanjing China; ^4^ Division of Communicable Disease Control Huai's Centre for Disease Control and Prevention Huaian China

**Keywords:** gene polymorphism, miRNA expression, transforming growth factor‐β1 receptor, type 2 diabetes mellitus

## Abstract

While the transforming growth factor‐β1 (TGF‐β1) regulates the growth and proliferation of pancreatic β‐cells, its receptors trigger the activation of Smad network and subsequently induce the insulin resistance. A case‐control was conducted to evaluate the associations of the polymorphisms of TGF‐β1 receptor‐associated protein 1 (*TGFBRAP1*) and TGF‐β1 receptor 2 (*TGFBR2*) with type 2 diabetes mellitus (T2DM), and its genetic effects on diabetes‐related miRNA expression. miRNA microarray chip was used to screen T2DM‐related miRNA and 15 differential expressed miRNAs were further validated in 75 T2DM and 75 normal glucose tolerance (NGT). The variation of rs2241797 (T/C) at *TGFBRAP1* showed significant association with T2DM in case‐control study, and the *OR* (95% *CI*) of dominant model for cumulative effects was 1.204 (1.060‐1.370), Bonferroni corrected *P* < 0.05. Significant differences in the fast glucose and HOMA‐β indices were observed amongst the genotypes of rs2241797. The expression of has‐miR‐30b‐5p and has‐miR‐93‐5p was linearly increased across TT, TC, and CC genotypes of rs2241797 in NGT,* P*_*trend*_ values were 0.024 and 0.016, respectively. Our findings suggest that genetic polymorphisms of *TGFBRAP1* may contribute to the genetic susceptibility of T2DM by mediating diabetes‐related miRNA expression.

## 1 INTRODUCTION

Type 2 diabetes mellitus (T2DM) is a long‐term metabolic disease characterized by high blood glucose and insulin resistance, and frequently accompanied by complications in cardiovascular diseases, renal failure, and visual damage.^1^, ^2^ Prevalence rates differ across Asian populations, with higher rate occurring in Chinese at 11.6%.^3^ Fast changes in lifestyles and ageing population in the past three decades had significant influence on the increased prevalence of T2DM in Chinese adults,^4^, ^5^ with special concerns regarding raised incidence in children and adolescents.^6^

T2DM is extensively evidenced to show a highly hereditary tendency.^7^, ^8^, ^9^ Multiple genetic risk variants have been identified from genome‐wide association studies (GWAS), which partially explained the genetic variability of T2DM.^10^, ^11^, ^12^ Yet, many susceptible polymorphisms remain uncovered.

Transforming growth factor beta 1 (TGF‐β1) regulates cellular communications in multiple cell types, including the growth and differentiation of pancreatic β cell which secrets insulin.^13^ TGF‐β1 also played a role in human neuroendocrine to induce the production of somatostatin (SST), while SST acts as a growth inhibitor.^14^ The TGF‐SST connection provides control of cell growth and potentially stimulates an autocrine feedback loop in diabetics.^14^, ^15^ In addition, insulin resistance (IR), as an important physiological marker of T2DM, is closely related to the impaired endothelium‐dependent vasodilation.^16^

The TGF‐β1/Smad signalling pathway was found to be involved in vascular development and epithelial remodelling.^17^ It is reasonable to infer the potential involvement of TGF‐β1‐related pathway in diabetes. Indeed, TGF‐β interacts with transmembrane receptors such as TGF‐β1 receptor 1 (TGFBR1), TGF‐β1 receptor 2 (TGFBR2), and TGF‐β1 receptor 3 (TGFBR3) to mediate its effects. Amongst these three receptors, only TGFBR2 can bind TGF‐β1, and then it recruits and phosphorylates TGFBR1.^18^ Animal experiments showed that TGFBR2 facilitated the cell differentiation and proliferation of β‐cells through the activation binding of Smad 2/3.^19^ TGF‐β receptor‐associated protein 1 (TGFBRAP1) was recently shown to be the molecular chaperone of Smad 4. It carries Smad 4 to the activated TGFBR2‐complex and promotes the phosphorylation of Smad 2/3, which subsequently induces the biological functions of the Smad network.^20^ Of particular interest to this study is the relevance of *TGFBR2* and *TGFBRAP1* polymorphisms to the genetic susceptibility of T2DM.

As a kind of noncoding RNA, microRNA (miRNA) is generated from endogenous hairpin structured transcripts throughout the genome and regulates at least 20%‐30% of all human genes by epigenetic modification.^21^ Specifically, miRNA involves in insulin secretion, β‐cell differentiation, glucolipid metabolism, and many other diabetes‐related processes^22^ and a number of studies have reported that miRNA contributes to the progression of T2DM.^23^, ^24^ To date, however, it has not been clarified whether the gene expression of *TGFBR2* and *TGFBRAP1* involving in the development of T2DM is stimulated or suppressed by miRNA‐binding target SNPs, or the variants at *TGFBR2* and *TGFBRAP1* contribute to its genetic effects on diabetes‐related miRNA expression by epigenetic modifications.

The purpose of this study is to investigate the associations of nine single nucleotide polymorphisms (SNPs) at *TGFBR2* and three SNPs at *TGFBRAP1* with T2DM and to evaluate its genetic effects on diabetes‐related miRNA expression. These would provide a novel insight to our better understanding on the TGF‐β1 pathway with diabetes.

## 2 MATERIALS AND METHODS

### 2.1 Study population

A total of 4222 subjects were recruited from a rural population in Yixing city (Jiangsu province, China), which had been described previously.^25^ The individuals were considered to be T2DM cases according to the presence of fasting plasma glucose (FPG) ≥7.0 mmol/L or a self‐reported T2DM history. Subjects with FPG between 5.6 and 6.9 mmol/L were defined to have impaired fasting glucose (IFG), and those with FPG <5.6 mmol/L normal glucose tolerance (NGT). After further verification with 3 months, a total of 468 T2DM cases and 899 IFG subjects were selected, excluding individuals with cardiovascular diseases, stroke, and cancer. Two thousand eight hundred and fifty‐five of age‐grouped (±2 years) and gender‐matched healthy individuals were identified as NGT controls.

The study protocol was approved by the Research Ethics Committee of Nanjing Medical University (NMU03307). All subjects were well informed about the current study and provided written consents; all methods were performed in accordance with the relevant guidelines and regulations.

### 2.2 Questionnaire survey and anthropometric measurement

The investigators were uniformly trained and qualified. All subjects completed a standard questionnaire including demographic characteristics, smoking and drinking habits, medical history, and underwent physical examinations including weight, height, and blood pressure (BP) by trained research staff. Body weight and height were measured twice for each individual without heavy clothes and shoes, and were rounded to the nearest 0.1 kg and 0.1 cm, respectively. Body mass index (BMI) was then calculated as weight (kg)/height squared (m^2^).

### 2.3 Chemical indices detection

Blood samples were collected after 8 hours from the last meal or during an overnight to measure fast glucose (GLU) using the glucose oxidase method. Insulin was detected using chemiluminescence while homeostatic model assessment of IR (HOMA‐IR) and HOMA of β‐cell functions (HOMA‐β) were calculated. HOMA‐IR = fasting plasma glucose × fasting plasma insulin/22.5, HOMA‐β = 20 × fasting plasma insulin/(fasting plasma glucose‐3.5).

### 2.4 miRNA isolation and detection

At the preliminary screening stage, total RNA was extracted in 600 μL plasma from 24 T2DM cases and 24 NGT respectively using miRNA microarray chip (Human microRNA Panelversion 1.0; Applied Biosystems, Foster City, CA). Specifically, 15 miRNAs were identified with the differential expression more than 2‐fold changed between T2DM and NGT. These 15 miRNAs were further validated in 75 T2DM cases and 75 NGT (Table S8).

RNA isolation was done using the NucleoSpin^®^ miRNA Plasma kit (MACHEREY‐NAGEL, Düren, Germany) according to the manufacturer's protocols. The concentration and purity of RNA samples were determined using a NanoDrop 2000 spectrophotometer (Thermo Fisher Scientific, Waltham, MA). The complementary DNA (cDNA) served as a template for miRNA quantitative PCR (qPCR) analysis was synthesized with TaqMan MicroRNA Reverse Transcription Kit (Applied Biosystems) with the Megaplex™ RT Reactions system. The thermal cycling parameters were 30 minutes at 16°C, 30 minutes at 42°C, and 5 minutes at 85°C.

The qPCR reaction was performed in triplicate to evaluate miRNA expression (5 μL reaction) in the plasma using the ABI RT‐PCR 7900 system (Applied Biosystems; Thermo Fisher Scientific, Inc.). The qPCR parameters were 10 minutes at 95°C followed by 40 cycles of 15 seconds at 95°C and 1 minute at 60°C. Cel‐miR‐39 was used as an endogenous control. The relative expression of miRNAs in plasma was calculated with comparative cycle threshold (ΔCT) method. The CT value >35 was considered to be undetectable data, and CT value ≤35 was normalized by the ΔCT method with cel‐miR‐39, which had a stable CT value in the plasma of two groups.

### 2.5 SNP selection and genotyping

The *TGFBR2* gene (gene ID: 7048) is located on chromosome 3 at p24.1 and spans 87.65 kbps. The *TGFBRAP1* gene (Gene ID: 9392) maps onto chromosome 2 at q12.1 and spans 80.29 kbps. We searched SNPs covering each of the genes starting from the upstream 2 kb to the downstream 1 kb and selected tagging SNPs (tagSNPs) from the database of International HapMAP Project. All the tagSNPs were selected with minor allele frequency (MAF) ≥0.05 and linkage disequilibrium (LD)—*r* ^2^ ≥ 0.8. Nine of *TGFBR2* SNPs (rs6785358, rs764522, rs9850060, rs3773645, rs749794, rs3773661, rs11709624, rs1155705, and rs1036096) and three of *TGFBRAP1* SNPs (rs17030766, rs2241797, and rs2679860) were examined in the current study (Table S1). SNP genotyping was performed using TaqMan technology (Applied Biosystems). All the genotype‐calling success rates were greater than 99.9%.

### 2.6 Statistical Analysis

The database was established in Epidata 3.0 (The Epidata Association, Odense, Denmark) and all statistical analyses were performed in SPSS version 15.0 (SPSS Inc., Chicago, IL). Qualitative variables amongst subject groups were compared using the Chi square (χ^*2*^) test and a two‐tailed *P* value of 0.05 was defined to be statistically significant. Fisher's exact test was used to test for HWE in the NGT group. Logistic regressions were applied to estimate the associations of the SNPs with IFG or T2DM and the odds ratio (OR) and 95% confidence intervals (CI) were calculated. Ordinal Logistic models were used to analyse the cumulative effects of the SNPs on IFG and T2DM. Cox proportional hazard model was applied to estimate the hazard ratio (HR) in the follow‐up study. Quantitative traits of fast blood glucose and insulin regulation including logarithmically transformed insulin (lginsulin), HOMA‐IR (lgHOMA‐IR) and HOMA‐β (lgHOMA‐β) amongst the genotypes were compared using a general linear model (GLM) with such confounding factors as age, gender, and BMI adjusted. The skewed distribution of miRNA level was transformed into normal distribution by the Box‐Cox model. The difference of miRNA expression between T2DM and NGT was found using Student's *t* test. Analysis of variance (ANOVA) was applied to compare the plasma miRNA expression amongst the three genotypes of SNPs. A two‐tailed *P *< 0.05 was considered statistically significant.

## 3 RESULTS

### 3.1 Demographic characteristics

In case‐control study, the demographic and clinical characteristics of participants were summarized in Table 1. No significant difference in gender ratio was found amongst T2DM, IFG, and NGT groups, with males accounting 36.2%, 41.2%, and 41.1%, respectively (*P *> 0.05). The ages of T2DM cases were slightly higher than those of the NGTs (+1.42 years) while BMI in both T2DM and IFG groups were significantly higher than in the NGTs. Thus, demographic characteristics of age, gender, and BMI were adjusted also before the genetic effects of SNPs were evaluated.

**Table 1:** Demographic characteristics of T2DM, IFG, and NGT subjects

Variables	Group	T2DM (n = 468)	IFG (n = 899)	NGT (n = 2855)	*F/*χ^*2*^	*P*
Gender	Male	163 (36.2%)	375 (41.2%)	1177 (41.1%)	4.04	0.133
	Female	287 (63.8%)	535 (58.8%)	1685 (58.9%)		
Age		61.61 ± 10.21	60.86 ± 10.89	60.29 ± 10.74	3.49	0.031
BMI (kg/m^2^)		25.07 ± 3.47	24.81 ± 3.45	23.83 ± 3.31	48.54	<0.001
GLU (mmol/L)		9.38 ± 3.27	6.03 ± 0.36	4.90 ± 0.59	2860.94	<0.001
LgInsulin		0.85 ± 0.34	0.76 ± 0.30	0.65 ± 0.32	95.04	<0.001
LgHOMA‐IR		0.45 ± 0.37	0.19 ± 0.31	‐0.01 ± 0.33	423.56	<0.001
LgHOMA‐β		1.44 ± 0.41	1.67 ± 0.30	1.82 ± 0.34	264.32	<0.001

T2DM, type 2 diabetes mellitus; IFG, impaired fasting glucose; NGT, normal glucose tolerance; BMI, body mass index; GLU, glucose; lgInsulin, logarithmically transformed insulin; lgHOMA‐IR, logarithmically transformed homeostatic model assessment (HOMA) of insulin resistance; lgHOMA‐β: logarithmically transformed HOMA of β‐cell functions.

### 3.2 Plasma levels of TGF‐β1 amongst subject groups

**Figure 1:**
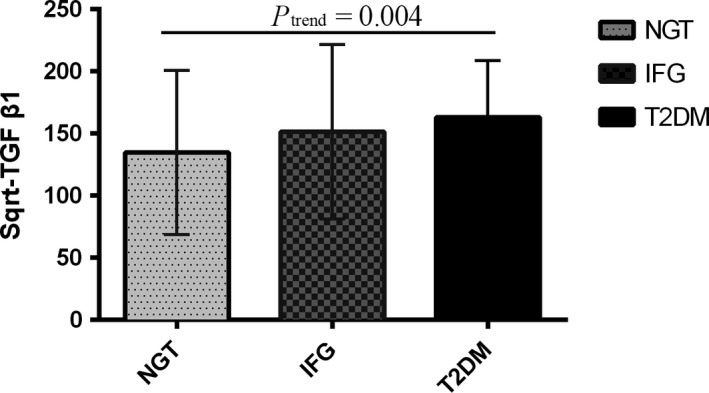
The Squared levels of plasma TGF‐β1 in the subject groups of NGT, IFG, and T2DM. There was a significant difference in the increasing trend of Sqrt‐TGFb1 amongst the groups (*P*_*trend*_ **= 0.004). Sqrt‐TGFb1 (mean ± SD) was the highest in the diabetics (134.92 ± 66.02), followed by subjects who had IFG (151.43 ± 70.18) and NGT (162.95 ± 45.86). T2DM, type 2 diabetes mellitus; IFG, impaired fasting glucose; NGT, normal glucose tolerance

There was significant difference in Sqrt‐TGF‐β1 concentration between T2DM, IFG and NGT groups with *P*_*trend*_ **= 0.004 (Figure 1). As expected, an increasing trend of TGF‐β1 plasma level was observed amongst subject groups. Sqrt‐TGF‐β1 (mean ± SD) was the highest in the diabetics (134.92 ± 66.02), followed by subjects who had IFG (151.43 ± 70.18) and NGT (162.95 ± 45.86).

### 3.3 Association analysis for T2DM and IFG

The allele frequency distributions of 12 SNPs were complied with Hardy‐Weinberg equilibrium. Ordinal logistic regression analyses displayed a significant association of dominant rs749794 at *TGFBR2* with T2DM and IFG, the OR (95% CI) was 1.146 (1.007‐1.304), *P *=* *0.038 (Table 2). The other eight SNPs showed no significant associations with T2DM or IFG (Table S2).

**Table 2:** Association analysis of *TGFBR2* and *TGFBRAP1* with IFG and T2DM

Gene	SNP	Group	WT/HT/MT	OR (95% CI)^a^, *P*		
				Additive	Dominant	Recessive
*TGFBR2*	rs749794		CC/CT/TT			
		NGT	1298/1235/322	Reference	Reference	Reference
		IFG	385/426/88	1.028 (0.918‐1.152), 0.628	1.114 (0.957‐1.298), 0.164	0.858 (0.668‐1.103), 0.233
		T2DM	191/221/56	1.131 (0.977‐1.309), 0.101	1.212 (0.992‐1.481), 0.059	1.078 (0.794‐1.464), 0.630
		Cumulative effect^b^		1.069 (0.972‐1.177), 0.170	1.146 (1.007‐1.304), 0.038	0.959 (0.780‐1.178), 0.690
*TGFBRAP1*	rs2241797		TT/TC/CC			
		NGT	1513/1126/220	Reference	Reference	Reference
		IFG	446/389/73	1.128 (1.002‐1.269), 0.046	1.192 (1.024‐1.387), 0.023	1.070 (0.811‐1.414), 0.631
		T2DM	215/194/39	1.152 (0.988‐1.343), 0.071	1.219 (1.000‐1.485), 0.049	1.117 (0.780‐1.598), 0.546
		Cumulative effect^b^		1.142 (1.034‐1.262), 0.009	1.204 (1.060‐1.370), 0.005	1.108 (0.877‐1.402), 0.387
	rs2679860		AA/AG/GG			
		NGT	1956/798/102	Reference	Reference	Reference
		IFG	611/271/27	1.048 (0.912‐1.203), 0.510	1.090 (0.929‐1.282), 0.289	0.849 (0.550‐1.310), 0.459
		T2DM	286/147/17	1.196 (1.005‐1.424), 0.044	1.274 (1.036‐1.565), 0.022	1.040 (0.614‐1.762), 0.883
		Cumulative effect b		1.110 (0.988‐1.246), 0.080	1.171 (1.013‐1.330), 0.032	0.952 (0.668‐1.358), 0.785

T2DM, type 2 diabetes mellitus; IFG, impaired fasting glucose; NGT, normal glucose tolerance; WT, wild‐type; HT, heterozygote; MT, mutant type; OR, odds ratio; CI, confidence interval; SNP, single nuclear polymorphisms.

Adjusted for age, gender, and BMI.

Cumulative effect was estimated by Ordinal logistic regression.

The cumulative effects of rs2241797 at *TGFBRAP1* demonstrated significant associations with T2DM and IFG in the additive model (OR, 95% CI: 1.142, 1.034‐1.262) and dominant model (OR, 95% CI: 1.204, 1.060‐1.370) with *P* values of 0.009 and 0.005, respectively (Table 2). The dominant model of rs2241797 was still sound after Bonferroni correction. The variation of rs2679860 at *TGFBRAP1* significantly increased the risk of T2DM, the OR (95% CI) for dominant model was 1.274 (1.036‐1.565), *P *= 0.022. Meanwhile, a significant association of the cumulative effect of rs2679860 with T2DM was also examined, and OR (95% CI) for dominant model was 1.171 (1.013‐1.330), *P *= 0.032. In addition, there was no association of rs17030766 at *TGFBRAP1* with T2DM and IFG.

### 3.4 Joint effect analysis of the positive SNPs with T2DM

Each pair from the three positive SNPs was fitted using Ordinal Logistic regression to assess the within‐pair independence, with two SNPs entering the Logistic regression model at the same time. The results indicated that SNP rs749794 had significant independent effect on T2DM with rs2241797 or rs2679860 while the effects of rs2241797 and rs2679860 were related (Table 3). A moderate LD (*r* ^2^ = 0.31) is found between rs2241797 and rs2679860 in the whole study population.

**Table 3:** Pairwise conditional and independent effect analyses for the three positive SNPs on T2DM risk

Independent SNP assumed	No.	SNPs in model	OR (95% CI)	*P*	Effect	D’/*r* ^2^
rs749794	1	rs749794	1.146 (1.007‐1.304)	0.038		
	2	rs749794	1.147 (1.008‐1.305)	0.038	Independent	
		rs2241797	1.205 (1.060‐1.370)	0.004		
	3	rs749794	1.145 (1.006‐1.303)	0.040	Independent	
		rs2679860	1.170 (1.022‐1.340)	0.023		
rs2241797	1	rs2241797	1.202 (1.058‐1.367)	0.005		
	2	rs2241797	1.073 (0.911‐1.264)	0.398	Related	0.31
		rs2679860	1.165 (0.998‐1.360)	0.054		
rs2679860	1	rs2679860	1.168 (1.020‐1.337)	0.025		

T2DM, type 2 diabetes mellitus; IFG, impaired fasting glucose; NGT, normal glucose tolerance; SNP, single nuclear polymorphisms; OR, odds ratio; CI, confidence interval; D’, standardized coefficient of linkage disequilibrium; *r* ^2^, correlation coefficient of allele frequencies of two loci.

The pairwise conditional and independent effects were estimated by Ordinal Logistic regression model as well as adjusting for age, sex, and body mass index; Independent effect was defined as both two SNPs presented lower D’/*r* ^2^ values with statistical significance (*P *<* *0.05) and related effect was defined as one SNP higher D’/*r* ^2^ values without statistical significance (*P *>* *0.05).

The joint effects of all three positive SNPs associated with T2DM were further analysed. rs2241797, which had a relative stronger risk than rs2679860, was selected to analyse the joint effect with rs749794 on T2DM and the results showed that the risk of T2DM increased significantly with the increase in number of risk alleles (*P*_*trend*_ = 0.004, Table 4). Compared with those without risk allele, individuals who carried one or more risk alleles had an overall 25.7% increased risk for T2DM.

**Table 4:** Joint effects of rs749794 and rs2241797 on the risk of T2DM

The number of effect allele	NGT (%)	IFG/T2DM (%)	*OR* (95% *CI*)	*P*
0	689 (24.2)	272 (20.0)	1	
1	1171 (41.1)	573 (42.0)	1.194 (1.006 − 1.418)	0.042
2‐4	992 (26.0)	518 (38.0)	1.154 (1.057 − 1.260)	0.001
Test for trend			1.110 (1.033 − 1.192)	*P*_*trend*_ = 0.004
0	689 (24.2)	272 (20.0)	1	
1‐4	2163 (75.8)	1091 (80.0)	1.257 (1.074 − 1.471)	0.004

T2DM: type 2 diabetes mellitus; IFG: impaired fasting glucose; NGT: normal glucose tolerance; SNP, single nuclear polymorphisms; OR, odds ratio; CI, confidence interval.

The number of effect allele was defined as the number of variant risk alleles of rs749794 and rs2241797 a person carries, null risk allele was taken as reference and the risks of *OR* (95% *CI*) for different number of effect allele were estimated by Ordinal Logistic regression model as well as adjustment for age, sex and body mass index. Furthermore, a trend effect of the allele rank was estimated for the cumulative increased risk and corresponding *P* for trend was calculated. Average risk was estimated for 1‐4 effect alleles of rs749794 and rs2241797.

### 3.5 Comparisons of quantitative traits amongst different genotypes of SNPs at *TGFBR2* and *TGFBRAP1*

Considering that antidiabetic drugs might affect the level of GLU, T2DM cases were divided into two groups of treatment and nontreatment (Table 5). GLM analyses showed in the IFG group, the level of GLU was significantly different amongst the genotypes of rs764522 at *TGFBR2*, and the *P* value was 0.047. Levels of lginsulin, lgHOMA‐IR, and lgHOMA‐β in the IFG group were significantly distinct amongst CC, CG, and GG genotype of rs3773645 at *TGFBR2*, with *P* values of 0.014, 0.018, and 0.009, respectively. Besides, in NGT group, the lginsulin was significantly different amongst three genotypes of rs3773645, and *P* value was 0.046. Levels of lginsulin and lgHOMA‐IR in the T2DM treatment group were significantly different amongst AA, AG, and GG genotype of rs6785358 at *TGFBR2*, with *P* values of 0.031 and 0.016, respectively.

**Table 5:** Quantitative traits comparisons amongst genotypes of *TGFBR2* and *TGFBRAP1* using multivariate ANOVA

Traits	SNPs	Genotype	NGT	IFG	T2DM (nontreatment)	T2DM (treatment)
GLU	rs764522	CC	4.89 ± 0.61 (n = 2130)	6.04 ± 0.36 (n = 677)	9.83 ± 3.23 (n = 254)	9.19 ± 3.44 (n = 122)
	(*TGFBR2*)	CG	4.94 ± 0.55 (n = 602)	6.01 ± 0.36 (n = 208)	9.48 ± 2.95 (n = 59)	8.63 ± 3.20 (n = 33)
		GG	5.03 ± 0.40 (n = 35)	6.31 ± 0.46 (n = 11)	8.01 ± 0.74 (n = 3)	9.36 (n = 1)
		*F*	2.640	3.070	0.692	0.264
		*P* a	0.072	0.047	0.501	0.769
	rs2241797	TT	4.88 ± 0.61 (n = 1510)	6.05 ± 0.37 (n = 438)	10.02 ± 3.30 (n = 1057)	9.14 ± 3.48 (n = 69)
	(*TGFBRAP1*)	TC	4.90 ± 0.59 (n = 1122)	6.03 ± 0.35 (n = 386)	9.55 ± 3.02 (n = 132)	9.01 ± 3.37 (n = 69)
		CC	5.01 ± 0.45 (n = 220)	5.99 ± 0.33 (n = 73)	9.49 ± 3.10 (n = 28)	9.09 ± 2.98 (n = 11)
		*F*	5.098^b^	0.396	0.508	0.046
		*P* a	0.006	0.673	0.602	0.955
Lginsulin	rs6785358	AA	0.66 ± 0.31 (n = 1932)	0.77 ± 0.30 (n = 617)	0.86 ± 0.34 (n = 237)	0.79 ± 0.35 (n = 110)
	(*TGFBR2*)	AG	0.67 ± 0.32 (n = 635)	0.77 ± 0.31 (n = 207)	0.84 ± 0.34 (n = 67)	0.90 ± 0.34 (n = 34)
		GG	0.60 ± 0.36 (n = 53)	0.76 ± 0.25 (n = 23)	0.97 ± 0.06 (n = 3)	0.99 (n = 1)
		*F*	0.912	0.087	0.367	3.562
		*P* a	0.402	0.917	0.693	0.031
	rs3773645	CC	0.67 ± 0.31 (n = 1237)	0.75 ± 0.31 (n = 386)	0.86 ± 0.32 (n = 135)	0.83 ± 0.33 (n = 74)
	(*TGFBR2*)	CG	0.64 ± 0.32 (n = 1183)	0.79 ± 0.30 (n = 373)	0.88 ± 0.34 (n = 145)	0.84 ± 0.38 (n = 55)
		GG	0.66 ± 0.32 (n = 281)	0.71 ± 0.29 (n = 91)	0.76 ± 0.37 (n = 30)	0.72 ± 0.37 (n = 17)
		*F*	3.089	4.291	1.416	1.199
		*P* a	0.046	0.014	0.244	0.304
LgHOMA‐IR	rs6785358	AA	−0.01 ± 0.325 (n = 1932)	0.19 ± 0.30 (n = 617)	0.48 ± 0.37 (n = 237)	0.37 ± 0.37 (n = 110)
	(*TGFBR2*)	AG	0.01 ± 0.337 (n = 635)	0.19 ± 0.31 (n = 207)	0.46 ± 0.38 (n = 67)	0.50 ± 0.36 (n = 34)
		GG	−0.06 ± 0.38 8(n = 53)	0.19 ± 0.25 (n = 23)	0.59 ± 0.12 (n = 3)	0.38 (n = 1)
		*F*	0.714	0.120	0.244	4.286
		*P* a	0.490	0.887	0.784	0.016
	rs9850060	AA	−0.01 ± 0.33 (n = 1716)	0.19 ± 0.30 (n = 524)	0.49 ± 0.35 (n = 200)	0.44 ± 0.38 (n = 87)
	(*TGFBR2*)	AG	−0.01 ± 0.34 (n = 854)	0.19 ± 0.31(n = 291)	0.47 ± 0.42(n = 99)	0.38 ± 0.37 (n = 50)
		GG	−0.04 ± 0.35 (n = 131)	0.23 ± 0.32 (n = 34)	0.39 ± 0.30 (n = 11)	0.19 ± 0.30 (n = 9)
		*F*	0.441	0.594	0.421	3.477^b^
		*P* a	0.643	0.552	0.657	0.034
	rs3773645	CC	−0.01 ± 0.33 (n = 1237)	0.18 ± 0.31 (n = 386)	0.48 ± 0.35 (n = 135)	0.40 ± 0.38 (n = 74)
	(*TGFBR2*)	CG	−0.03 ± 0.34 (n = 1183)	0.22 ± 0.30 (n = 373)	0.51 ± 0.38 (n = 145)	0.43 ± 0.39 (n = 55)
		GG	−0.01 ± 0.34 (n = 281)	0.14 ± 0.3 (n = 91)	0.36 ± 0.38 (n = 30)	0.35 ± 0.33 (n = 17)
		*F*	2.545	4.014	1.256	0.873
		*P* a	0.079	0.018	0.286	0.420
	rs749794	CC	−0.01 ± 0.34 (n = 1223)	0.19 ± 0.31 (n = 363)	0.43 ± 0.32 (n = 126)	0.45 ± 0.39 (n = 59)
	(*TGFBR2*)	CT	−0.01 ± 0.33 (n = 1179)	0.20 ± 0.30 (n = 404)	0.49 ± 0.39 (n = 146)	0.34 ± 0.37 (n = 71)
		TT	−0.05 ± 0.33 (n = 302)	0.17 ± 0.34 (n = 83)	0.57 ± 0.43 (n = 38)	0.55 ± 0.27 (n = 16)
		*F*	0.632	0.226	3.328	1.446
		*P* a	0.532	0.798	0.037	0.239
	rs1036096	CC	−0.01 ± 0.33 (n = 946)	0.18 ± 0.30(n = 290)	0.50 ± 0.36(n = 101)	0.45 ± 0.41(n = 66)
	(*TGFBR2*)	CT	−0.02 ± 0.34 (n = 1281)	0.20 ± 0.31 (n = 407)	0.50 ± 0.39 (n = 151)	0.35 ± 0.37 (n = 64)
		TT	−0.01 ± 0.33 (n = 477)	0.20 ± 0.31 (n = 153)	0.36 ± 0.33 (n = 58)	0.43 ± 0.25 (n = 16)
		*F*	1.948	0.039	3.284	1.152
		*P* a	0.143	0.961	0.039	0.319
LgHOMA‐β	rs3773645	CC	1.83 ± 0.34 (n = 1200)	1.65 ± 0.30 (n = 386)	1.42 ± 0.39 (n = 135)	1.50 ± 0.43 (n = 74)
	(*TGFBR2*)	CG	1.81 ± 0.35 (n = 1156)	1.69 ± 0.30 (n = 373)	1.42 ± 0.36 (n = 145)	1.46 ± 0.46 (n = 55)
		GG	1.83 ± 0.34 (n = 276)	1.61 ± 0.29 (n = 91)	1.35 ± 0.43 (n = 30)	1.26 ± 0.49 (n = 17)
		*F*	2.282	4.732	1.184	2.133
		*P* a	0.102	0.009	0.307	0.122
	rs749794	CC	1.82 ± 0.34 (n = 1192)	1.67 ± 0.3 (n = 363)	1.40 ± 0.34 (n = 126)	1.39 ± 0.51 (n = 59)
	(*TGFBR2*)	CT	1.83 ± 0.34 (n = 1151)	1.67 ± 0.3 (n = 404)	1.40 ± 0.42 (n = 146)	1.43 ± 0.38 (n = 71)
		TT	1.79 ± 0.36 (n = 292)	1.63 ± 0.34 (n = 83)	1.49 ± 0.32 (n = 38)	1.80 ± 0.39 (n = 16)
		*F*	0.668	0.368	0.606	5.412^b^
		*P* a	0.513	0.692	0.546	0.005
	rs2241797	TT	1.83 ± 0.35 (n = 1403)	1.67 ± 0.31 (n = 420)	1.40 ± 0.38 (n = 150)	1.42 ± 0.44 (n = 68)
	(*TGFBRAP1*)	TC	1.81 ± 0.32 (n = 1025)	1.65 ± 0.29 (n = 362)	1.41 ± 0.34 (n = 130)	1.47 ± 0.46 (n = 69)
		CC	1.77 ± 0.35 (n = 205)	1.65 ± 0.22 (n = 67)	1.42 ± 0.47 (n = 28)	1.52 ± 0.37 (n = 9)
		*F*	3.188^b^	0.488	0.120	0.051
		*P* a	0.041	0.614	0.887	0.950

T2DM, type 2 diabetes mellitus; IFG, impaired fasting glucose; NGT, normal glucose tolerance; SNP, single nuclear polymorphisms.

a The comparisons of quantitative traits amongst genotypes of *TGFBR2* and *TGFBRAP1* after adjustment for age, gender, and BMI.

b *P *<* *0.05 for trend test.

The lgHOMA‐IR gradually decreased across AA, AG, and GG genotype of rs9850060 at *TGFBR2* in the T2DM treatment group, with a *P*_*trend*_ value of 0.013. Significant differences in lgHOMA‐IR amongst the CC, CT, and TT genotype of rs749794 at *TGFBR2* were observed in T2DM treatment, *P *= 0.037. Meanwhile, in the nontreatment group, the lgHOMA‐β levels gradually elevated across the three genotypes of rs749794, with a *P*_*trend*_ value of 0.005. Additionally, the lgHOMA‐IR was significantly different amongst CC, CT, and TT genotype of rs1036096 at *TGFBR2* in T2DM nontreatment group, *P *= 0.039.

The level of GLU gradually increased across TT, TC, and CC genotypes of rs2241797 at *TGFBRAP1* in NGT group, with a *P*_*trend*_ value of 0.006, while an inverse trend for lgHOMA‐β was observed (*P*_*trend*_ **= 0.041). The quantitative traits of all selected SNPs were listed in Table S3‐S6.

### 3.6 Comparison of miRNA expression amongst genotypes of *TGFBR2* and *TGFBRAP1*

**Figure 2:**
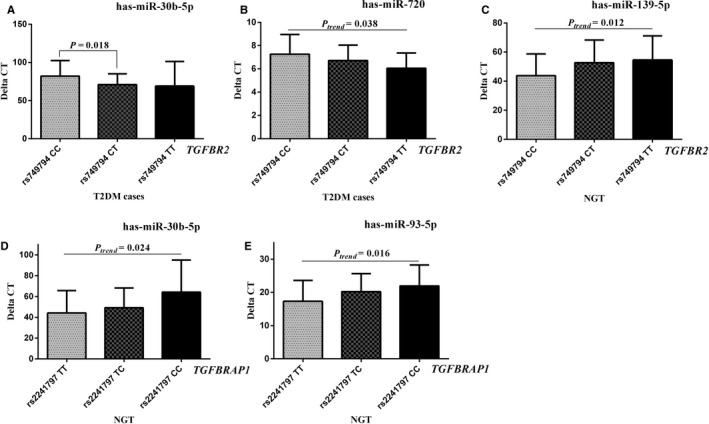
Comparison of miRNA expression amongst different genotypes in T2DM and NGT. Significant different expressions for has‐miR‐30b‐5p and has‐miR‐720 were observed in T2DM cases with rs749794 CC, CT and TT genotype (A and B); while the has‐miR‐139‐5p had an elevated trend amongst rs749794 CC, CT and TT in NGT (C). The expression of has‐miR‐30b‐5p and has‐miR‐93‐5p was significantly increased across TT, TC and CC genotypes of rs2241797, *P*_*trend*_ values were 0.024 and 0.016, respectively (D and E). T2DM, type 2 diabetes mellitus; IFG, impaired fasting glucose; NGT, normal glucose tolerance

A total of 12 miRNAs presented differential expression between T2DM cases and controls (Table S7). The expression of has‐miR‐30b‐5p was found to be significantly different amongst the genotypes of rs749794 in T2DM, *P *= 0.041; post hoc multiple comparisons showed T2DM subjects with rs749794 CC genotype had a high level of has‐miR‐30b‐5p than CT carriers, *P *= 0.018. The expression of has‐miR‐720 was significantly decreased across rs749794 CC, CT and TT genotypes, with a *P*_*trend*_ value of 0.038. In controls, the expression of has‐miR‐139‐5p gradually elevated across rs749794 CC, CT and TT carriers, with a *P*_*trend*_ value of 0.043. The expression of has‐miR‐30b‐5p and has‐miR‐93‐5p was significantly increased across TT, TC, and CC genotype of rs2241797, *P*_*trend*_ values were 0.024 and 0.016, respectively (Figure 2). These results were also listed in Table S8.

## 4 DISCUSSION

TGF‐β1 plays a vital role in regulating the growth and proliferation of pancreatic β cells which are responsible for the insulin secretion.^14^ Although the distinct role of TGFBR2 and TGFBRAP1 in the TGF‐β1/SMAD signalling pathway had been observed previously,^18^, ^20^ no genetic association study was conducted to evaluate the correlation of *TGFRB2* and *TGFBRAP1* polymorphisms with T2DM. The current study firstly adopted a function candidate strategy to investigate the relevance of *TGFBR2* and *TGFBRAP1* polymorphisms to the genetic susceptibility to T2DM.

The results of this study replicated the relationship between TGF‐β1 and T2DM in the Chinese population and further confirmed the increasing levels of plasma TGF‐β1 in the individuals with T2DM followed by those with IFG compared to NGT subjects. For the first time, it was found that rs2241797 of *TGFBRAP1* significantly increased the risk of T2DM. The level of GLU was linearly increased amongst rs2241797 TT, TC and CC genotypes, while a decreased trend of HOMA‐β was observed in NGT individuals. The functional prediction of rs2241797 T>C notes that this missense variation overlaps with an enhancer (ESDR) and disrupts B double prime 1 (BDP1) motif.^26^ As the expression of has‐miR‐30b‐5p and has‐miR‐93‐5p were significantly increased across rs2241797 TT, TC and CC genotype, further functional experiment is warranted to illuminate whether rs2214797 affects the susceptibility to T2DM through the epigenetic mechanism.

The joint effect of rs2241797 and rs749794 on T2DM was identified as an allele number ranked dose‐response, which would be helpful to understand the molecular pathogenesis mechanism of T2DM, and to provide a scientific research basis for individualized drug therapy of patients with T2DM. Meanwhile, the plots of rs2241797 and rs749794 observed less medium to high LD (*r* ^2^ > 0.6) SNPs, which makes them more available to the selection of biofunctional research and prediction of T2DM.

An A>G mutation of rs2679860 located downstream of the *TGFBRAP1* gene would result in a negative influence on the combining functions of the transcriptional factors GCM and GATA‐1. The *TGFBRAP1* gene expression might therefore be modified and contribute to the risk of T2DM; however, the absence of its association with the quantitative traits of GLU might indicate an actual involvement of its closely related SNPs. Furthermore, we make a regional LD plot (http://www.broadinstitute.org/mpg/snap/ldplot.php) for the three positive SNPs (Figure S1). Several estimated loci near rs2679860 with high LD (*r* ^*2*^>0.8) suggest a fine mapping and further functional researches are necessary to evaluate the genetic effect of *TGFBRAP1* on T2DM.

The cumulative effect of rs749794 variation at *TGFBR2* was significantly associated with T2DM, and also displayed significant differences in the HOMA indexes for T2DM cases either with or without the antidiabetic treatment. The quantitative association of HOMA‐β and HOMA‐IR suggested that rs749794 might play an important role in regulating the insulin resistance and β‐cell functions.

In the current study, 12 miRNAs expressions were found to be significantly different in T2DM and NGT subjects. Four miRNAs (has‐miR‐30b‐5p, has‐miR‐93‐5p, has‐miR‐126‐3p, and has‐miR‐320a)^27^, ^28^, ^29^, ^30^ were further validated with previous studies; five miRNAs (has‐miR‐150‐5p, has‐miR‐328‐3p, has‐miR‐335‐5p, has‐miR‐511‐5p, and has‐miR‐720) were firstly demonstrated to be differentially expressed in T2DM and NGT, which might become new biomarkers for T2DM diagnosis. Nevertheless, three miRNAs expressions were contradictory with our findings. The level of has‐miR‐139‐5p was found to be significantly higher in T2DM than that in NGT in this study. However, it was also illustrated that no differential expression of has‐miR‐139‐5p in 55 T2DM patients and 80 controls, the conflicting result appeared due to the various tissues (microparticles) for miRNA isolation.^31^ Meanwhile, higher levels of has‐miR‐191‐5p and has‐miR‐574‐5p in T2DM cases were observed, which were inconsistent with previous studies.^32^, ^33^ This could be linked to the population studied, with ethnicities, age or gender difference considered.^34^ However, whether the expression of miRNA is ethnicity related in T2DM is not totally elucidated.

Functional studies have identified that increased miR‐30b level contributes to cytokine‐mediated β‐cell dysfunction occurring during the development and progression of type 1 diabetes.^27^ The miR‐93 expression was higher in the diabetic retinopathy group than those in the healthy group, and severed as a diagnostic marker for type 2 diabetic retinopathy. The current study showed that has‐miR‐30b‐5p and has‐miR‐93‐5p were elevated in T2DM compared with NGT, which were consistent with previous reports.^27^, ^35^ Specifically, we evaluated the effect of rs2241797 on these miRNAs expression, and has‐miR‐30b‐5p and has‐miR‐93‐5p were significantly increased across rs2241797 genotypes. Besides, the has‐miR‐30b‐5p and has‐miR‐720 expression were significantly distinct amongst rs749794 variation in T2DM, while has‐miR‐139‐5p expression gradually increased amongst rs749794 variation in NGT. These results indicate rs2241797 and rs749794 may contribute to the genetic susceptibility to T2DM by mediating diabetes‐related miRNA expression. This further favours the potential role of rs2241797 participating in the molecular mechanism of T2DM.

Several limitations in the present study were acknowledged. First, all participants were from the same area (Han population in south China) so that the subject diversity of varied cultures and lifestyles was limited. Second, serum TGFBRAP1 and TGFBR2 levels were not yet detected in our study. Finally, the association analyses would be better refined in a larger sample size.

To the authors’ best knowledge, the effects of genetic variants related to TGF‐β1 on the susceptibility of diabetes and related diseases have not been documented. The current study presents the novel and original findings that *TGFBRAP1* SNP rs2241797 was significantly associated with T2DM. The mutations were also found to be correlated with the quantitative characters of GLU, insulin, HOMA‐IR and HOMA‐β in NGT or IFG population. In addition, the expression of has‐miR‐30b‐5p and has‐miR‐93‐5p was significantly different amongst rs2241797 genotypes. This indicated that *TGFBRAP1* might participate in the epigenetic mechanism of diabetes; however, a further systemic functional analysis would be warranted and future studies of population diversity are desired.

In conclusion, our findings suggest that genetic polymorphisms of *TGFBRAP1* may contribute to the genetic susceptibility to T2DM by mediating diabetes‐related miRNA expression.

## AUTHOR CONTRIBUTION

Chong Shen designed this study. Song Yang, Xianghai Zhao and Yanchun Chen collected the data. Hailong Zhao accomplished laboratory work. Xiaotian Chen and Chong Shen analysed data. Mengyao Yang wrote the main manuscript text. Chunlan Liu and Chong Shen revised it.

## COMPETING INTERESTS

The authors confirm that there are no conflicts of interest.
