# Supplementary material for: The variant at TGFBRAP1 is significantly associated with type 2 diabetes mellitus and affects diabetes‐related miRNA expression
Source: J Cell Mol Med. 2018 Nov 20;23(1):83–92. doi: 10.1111/jcmm.13885 (PMC6307842; doi:10.1111/jcmm.13885)
Supplement: Supplementary file 1 [file JCMM-23-83-s001.docx]

**Polymorphism of Transforming Growth Factor-β1 Receptors Associated with Type**

**2 Diabetes Mellitus in Chinese Population**

Song Yang, Xiaotian Chen, Mengyao Yang, Xianghai Zhao, Yanchun Chen, Hailong Zhao, Chunlan Liu, Chong Shen^*^

**Table S1. The biological information and function prediction of selected tagSNPs**

| Gene | SNP | Chromosome | dbSNP func annot | Allele | TFBS | Motifs changed | RegPotential | Conservation | Nearby Gene |
| --- | --- | --- | --- | --- | --- | --- | --- | --- | --- |
| *TGFBR2* | rs1036096 | 3 | intronic | G/A | -- | GR,NRSF,Rad21 | NA | 0.002 | TGFBR2 |
|  | rs1155705 | 3 | intronic | A/G | -- | -- | 0.1794 | 0 | TGFBR2 |
|  | rs11709624 | 3 | intronic | C/G | -- | Nr2f2,Pbx-1 | 0 | 0 | TGFBR2 |
|  | rs3773645 | 3 | intronic | C/G | -- | -- | 0 | 0.003 | TGFBR2 |
|  | rs3773661 | 3 | intronic | C/G | -- | -- | 0 | 0 | TGFBR2 |
|  | rs6785358 | 3 | 5'UTR | A/G | HNF-3b | Nkx3,SRF,TATA | 0 | 0.001 | LOC100131843\|\|TGFBR2 |
|  | rs749794 | 3 | intronic | G/A | -- | -- | NA | 0.003 | TGFBR2 |
|  | rs764522 | 3 | 5'UTR | C/G | HSF | AP-1,Bach2 | 0.156073 | 0 | LOC100131843\|\|TGFBR2 |
|  | rs9850060 | 3 | intronic | A/G | -- | Pou2f2,Sox | 0 | 0.003 | TGFBR2 |
| *TGFBRAP1* | rs17030766 | 2 | intronic | A/G | -- | Bcl6b,Ik-1 | 0 | 0 | TGFBRAP1 |
|  | rs2241797 | 2 | missense | C/T | -- | -- | 0.488944 | 0 | TGFBRAP1 |
|  | rs2679860 | 2 | intronic | T/C | -- | COMP1,Gcm1 | 0 | 0.001 | TGFBRAP1 |

SNP, single nucleotide polymorphism; TFBS, transcription factor binding site; RegPotential, regulatory potential score.

**Table S2. Association analysis of *TGFBR2* and *TGFBRAP1* with IFG and T2DM**

| Gene | SNP | Group | WT/HT/MT | *OR* (95% *CI*)^a^, *P* | | |
| --- | --- | --- | --- | --- | --- | --- |
|  |  |  |  | Additive | Dominant | Recessive |
| *TGFBR2* | rs6785358 |  | AA/AG/GG |  |  |  |
|  |  | NGT | 2038/675/54 | Reference | Reference | Reference |
|  |  | IFG | 645/226/25 | 1.119(0.963-1.301), 0.143 | 1.100(0.928-1.304), 0.272 | 1.509(0.929-2.451), 0.096 |
|  |  | T2DM | 354/106/4 | 0.856(0.692-1.059), 0.151 | 0.873(0.692-1.100), 0.250 | 0.464(0.167-1.294), 0.142 |
|  |  | Cumulative effect ^b^ | | 0.996(0.874-1.135), 0.950 | 0.993(0.858-1.149), 0.923 | 1.018(0.642-1.614), 0.939 |
|  | rs764522 |  | CC/CG/GG |  |  |  |
|  |  | NGT | 2130/602/35 | Reference | Reference | Reference |
|  |  | IFG | 677/208/11 | 1.080(0.917-1.271), 0.359 | 1.094(0.916-1.307), 0.319 | 0.911(0.764-1.087), 0.303 |
|  |  | T2DM | 371/90/4 | 0.857(0.682-1.078), 0.187 | 0.855(0.669-1.092), 0.210 | 1.156(0.906-1.474), 0.243 |
|  |  | Cumulative effect ^b^ | | 0.979(0.850-1.128), 0.772 | 0.985(0.845-1.149), 0.850 | 1.009(0.866-1.175), 0.913 |
|  | rs9850060 |  | AA/AG/GG |  |  |  |
|  |  | NGT | 1819/894/138 | Reference | Reference | Reference |
|  |  | IFG | 559/303/36 | 1.024(0.898-1.166), 0.726 | 1.069(0.914-1.250), 0.403 | 0.832(0.570-1.214), 0.340 |
|  |  | T2DM | 296/152/20 | 1.003(0.845-1.190), 0.973 | 1.024(0.835-1.257), 0.817 | 0.899(0.554-1.458), 0.665 |
|  |  | Cumulative effect ^b^ | | 1.011(0.905-1.130), 0.842 | 1.044(0.915-1.192), 0.521 | 0.860(0.627-1.180), 0.350 |
|  | rs3773645 |  | CC /CG/GG |  |  |  |
|  |  | NGT | 1313/1248/291 | Reference | Reference | Reference |
|  |  | IFG | 409/395/95 | 1.020(0.910-1.144), 0.729 | 1.026(0.881-1.194), 0.743 | 1.028(0.803-1.315), 0.829 |
|  |  | T2DM | 216/204/48 | 0.996(0.858-1.156), 0.954 | 0.996(0.818-1.214), 0.972 | 0.989(0.715-1.368), 0.946 |
|  |  | Cumulative effect ^b^ | | 1.007(0.914-1.109), 0.893 | 1.010(0.888-1.148), 0.883 | 1.006(0.815-1.240), 0.958 |
|  | rs749794 |  | CC /CT/TT |  |  |  |
|  |  | NGT | 1298/1235/322 | Reference | Reference | Reference |
|  |  | IFG | 385/426/88 | 1.028(0.918-1.152), 0.628 | 1.114(0.957-1.298), 0.164 | 0.858(0.668-1.103), 0.233 |
|  |  | T2DM | 191/221/56 | 1.131(0.977-1.309), 0.101 | 1.212(0.992-1.481), 0.059 | 1.078(0.794-1.464), 0.630 |
|  |  | Cumulative effect ^b^ | | 1.069(0.972-1.177), 0.170 | 1.146(1.007-1.304), 0.038 | 0.959(0.780-1.178), 0.690 |
|  | rs3773661 |  | GG/GC/CC |  |  |  |
|  |  | NGT | 1346/1246/263 | Reference | Reference | Reference |
|  |  | IFG | 418/396/85 | 1.019(0.907-1.145), 0.751 | 1.028(0.884-1.196), 0.720 | 1.013(0.782-1.312), 0.925 |
|  |  | T2DM | 226/202/40 | 0.954(0.818-1.111), 0.542 | 0.954(0.783-1.162), 0.641 | 0.905(0.637-1.284), 0.575 |
|  |  | Cumulative effect ^b^ | | 0.991(0.898-1.093), 0.853 | 0.994(0.875-1.130), 0.931 | 0.971(0.778-1.211), 0.792 |
|  | rs11709624 |  | GG/GC/CC |  |  |  |
|  |  | NGT | 1394/1201/255 | Reference | Reference | Reference |
|  |  | IFG | 448/385/64 | 0.948(0.842-1.067), 0.373 | 0.974(0.837-1.133), 0.736 | 0.808(0.606-1.077), 0.145 |
|  |  | T2DM | 239/185/44 | 0.976(0.837-1.139), 0.760 | 0.930(0.763-1.132), 0.469 | 1.108(0.789-1.556), 0.554 |
|  |  | Cumulative effect ^b^ | | 0.960(0.868-1.061), 0.421 | 0.954(0.840-1.085), 0.476 | 0.935(0.741-1.180), 0.570 |
|  | rs1155705 |  | GG/GA/AA |  |  |  |
|  |  | NGT | 1351/1216/287 | Reference | Reference | Reference |
|  |  | IFG | 417/392/89 | 1.020(0.909-1.144), 0.734 | 1.036(0.891-1.206), 0.643 | 0.997(0.774-1.283), 0.979 |
|  |  | T2DM | 203/217/48 | 1.102(0.950-1.278), 0.200 | 1.166(0.956-1.423), 0.129 | 1.047(0.756-1.450), 0.783 |
|  |  | Cumulative effect ^b^ | | 1.054(0.957-1.161), 0.287 | 1.087(0.956-1.236), 0.201 | 1.023(0.827-1.265), 0.837 |
|  | rs1036096 |  | CC/CT/TT |  |  |  |
|  |  | NGT | 997/1358/500 | Reference | Reference | Reference |
|  |  | IFG | 305/433/161 | 1.024(0.920-1.140), 0.667 | 1.041(0.888-1.221), 0.621 | 1.018(0.835-1.240), 0.861 |
|  |  | T2DM | 172/220/76 | 0.934(0.811-1.075), 0.341 | 0.920(0.749-1.129), 0.422 | 0.903(0.692-1.178), 0.452 |
|  |  | Cumulative effect ^b^ | | 0.980(0.895-1.074), 0.667 | 0.980(0.857-1.121), 0.768 | 0.964(0.814-1.142), 0.672 |
| *TGFBRAP1* | rs17030766 |  | GG/GA/AA |  |  |  |
|  |  | NGT | 1878/883/98 | Reference | Reference | Reference |
|  |  | IFG | 613/265/30 | 0.936(0.815-1.076), 0.354 | 0.916(0.780-1.077), 0.288 | 0.992(0.652-1.508), 0.968 |
|  |  | T2DM | 290/140/20 | 1.083(0.909-1.290), 0.375 | 1.070(0.871-1.314), 0.521 | 1.292(0.787-1.508), 0.312 |
|  |  | Cumulative effect ^b^ | | 0.996(0.887-1.120), 0.950 | 0.980(0.895-1.074), 0.785 | 1.137(0.807-1.600), 0.463 |
|  | rs2241797 |  | TT/TC/CC |  |  |  |
|  |  | NG | 1513/1126/220 | Reference | Reference | Reference |
|  |  | IFG | 446/389/73 | 1.128(1.002-1.269), 0.046 | 1.192(1.024-1.387), 0.023 | 1.070(0.811-1.414), 0.631 |
|  |  | T2DM | 215/194/39 | 1.152(0.988-1.343), 0.071 | 1.219(1.000-1.485), 0.050 | 1.117(0.780-1.598), 0.546 |
|  |  | Cumulative effect ^b^ | | 1.142(1.034-1.262), 0.009 | 1.204(1.060-1.370), 0.005 | 1.108(0.877-1.402), 0.387 |
|  | rs2679860 |  | AA/AG/GG |  |  |  |
|  |  | NG | 1956/798/102 | Reference | Reference | Reference |
|  |  | IFG | 611/271/27 | 1.048(0.912-1.203), 0.510 | 1.090(0.929-1.282), 0.289 | 0.849(0.550-1.310), 0.459 |
|  |  | T2DM | 286/147/17 | 1.196(1.005-1.424), 0.044 | 1.274(1.036-1.565), 0.022 | 1.040(0.614-1.762), 0.883 |
|  |  | Cumulative effect ^b^ | | 1.110(0.988-1.246), 0.080 | 1.171(1.013-1.330), 0.032 | 0.952(0.668-1.358), 0.785 |

T2DM: type 2 diabetes mellitus; IFG: impaired fasting glucose; NGT: normal glucose tolerance; SNP, single nuclear polymorphisms; WT: wild type;

HT: heterozygote; MT: mutant type; OR, odds ratio; CI, confidence interval.

a. Adjusted for age, gender, and BMI

b. Cumulative effect was estimated by Ordinal logistic regression

**Table S3. Comparisons of fast glucose amongst genotypes of *TGFBR2* and *TGFBRAP1***

| Gene | SNPs | Genotype | NGT | IFG | T2DM (non-treatment) | T2DM (treatment) |
| --- | --- | --- | --- | --- | --- | --- |
| *TGFBR2* | rs6785358 | AA | 4.89±0.60(n=2038) | 6.03±0.35(n=645) | 9.77±3.22(n=243) | 8.91±3.31(n=116) |
|  |  | AG | 4.94±0.59(n=675) | 6.04±0.37(n=226) | 9.72±3.07(n=70) | 9.41±3.24(n=38) |
|  |  | GG | 4.96±0.65(n=54) | 6.10±0.41(n=25) | 9.48±1.34(n=3) | 5.48(n=1) |
|  |  | *F* | 1.820 | 0.402 | 0.083 | 1.133 |
|  |  | *P^a^* | 0.162 | 0.669 | 0.920 | 0.325 |
|  | rs764522 | CC | 4.89±0.61(n=2130) | 6.04±0.36(n=677) | 9.83±3.23(n=254) | 9.19±3.44(n=122) |
|  |  | CG | 4.94±0.55(n=602) | 6.01±0.36(n=208) | 9.48±2.95(n=59) | 8.63±3.20(n=33) |
|  |  | GG | 5.03±0.40(n=35) | 6.31±0.46(n=11) | 8.01±0.74(n=3) | 9.36(n=1) |
|  |  | *F* | 2.640 | 3.070 | 0.692 | 0.264 |
|  |  | *P^a^* | 0.072 | 0.047 | 0.501 | 0.769 |
|  | rs9850060 | AA | 4.90±0.60(n=1819) | 6.05±0.36(n=559) | 9.58±2.80(n=207) | 9.48±3.57(n=89) |
|  |  | AG | 4.89±0.57(n=894) | 6.01±0.35(n=303) | 10.13±3.85(n=101) | 8.47±3.13(n=51) |
|  |  | GG | 4.85±0.62(n=138) | 6.06±0.39(n=36) | 9.81±2.43(n=11) | 8.39±1.94(n=9) |
|  |  | *F* | 0.459 | 1.768 | 0.648 | 2.067 |
|  |  | *P^a^* | 0.632 | 0.171 | 0.524 | 0.130 |
|  | rs3773645 | CC | 4.89±0.62(n=1313) | 6.03±0.36(n=409) | 9.70±3.20(n=141) | 8.89±3.36(n=75) |
|  |  | CG | 4.9±0.56(n=1248) | 6.03±0.37(n=395) | 9.95±3.17(n=147) | 9.08±3.56(n=57) |
|  |  | GG | 4.91±0.57(n=291) | 6.06±0.34(n=95) | 9.26±2.94(n=31) | 9.87±2.77(n=17) |
|  |  | *F* | 0.258 | 0.372 | 0.249 | 0.706 |
|  |  | *P^a^* | 0.773 | 0.689 | 0.780 | 0.496 |
|  | rs749794 | CC | 4.91±0.59(n=1298) | 6.04±0.36(n=385) | 9.36±2.66(n=130) | 9.73±3.62(n=61) |
|  |  | CT | 4.89±0.57(n=1235) | 6.03±0.36(n=426) | 10.08±3.39(n=150) | 8.77±3.21(n=71) |
|  |  | TT | 4.87±0.65(n=322) | 6.06±0.39(n=88) | 9.90±3.65(n=39) | 7.89±2.72(n=17) |
|  |  | *F* | 0.851 | 0.438 | 2.151 | 2.813 |
|  |  | *P^a^* | 0.427 | 0.645 | 0.118 | 0.063 |
|  | rs3773661 | GG | 4.91±0.57(n=1346) | 6.03±0.36(n=418) | 9.75±3.24(n=162) | 8.73±3.21(n=64) |
|  |  | GC | 4.88±0.61(n=1246) | 6.05±0.36(n=396) | 9.87±3.08(n=131) | 9.29±3.45(n=71) |
|  |  | CC | 4.89±0.61(n=263) | 6.02±0.35(n=85) | 9.32±3.15(n=26) | 9.67±3.87(n=14) |
|  |  | *F* | 1.061 | 0.427 | 0.152 | 0.165 |
|  |  | *P^a^* | 0.346 | 0.653 | 0.859 | 0.848 |
|  | rs11709624 | GG | 4.91±0.59(n=1394) | 6.04±0.35(n=448) | 9.55±2.84(n=172) | 9.46±3.33(n=67) |
|  |  | GC | 4.89±0.58(n=1201) | 6.04±0.38(n=385) | 10.01±3.61(n=122) | 8.86±3.57(n=63) |
|  |  | CC | 4.88±0.65(n=255) | 6.00±0.34(n=64) | 10.01±2.97(n=25) | 8.41±2.75(n=19) |
|  |  | *F* | 0.465 | 0.139 | 0.706 | 1.397 |
|  |  | *P^a^* | 0.628 | 0.870 | 0.494 | 0.251 |
|  | rs1155705 | GG | 4.90±0.61(n=1351) | 6.05±0.37(n=417) | 9.57±2.80(n=146) | 9.44±3.55(n=57) |
|  |  | GA | 4.89±0.57(n=1216) | 6.03±0.35(n=392) | 9.75±3.27(n=143) | 8.98±3.29(n=74) |
|  |  | AA | 4.87±0.59(n=287) | 5.99±0.37(n=89) | 10.69±4.05(n=30) | 8.26±3.12(n=18) |
|  |  | *F* | 0.172 | 0.916 | 2.128 | 0.831 |
|  |  | *P^a^* | 0.842 | 0.401 | 0.121 | 0.438 |
|  | rs1036096 | CC | 4.90±0.61(n=997) | 6.03±0.36(n=305) | 9.77±3.47(n=105) | 9.18±3.39(n=67) |
|  |  | CT | 4.90±0.56(n=1358) | 6.05±0.36(n=433) | 9.95±3.13(n=155) | 8.70±3.43(n=65) |
|  |  | TT | 4.88±0.63(n=500) | 6.02±0.35(n=161) | 9.23±2.51(n=59) | 10.17±2.95(n=17) |
|  |  | *F* | 0.440 | 0.095 | 1.650 | 1.648 |
|  |  | *P^a^* | 0.644 | 0.909 | 0.194 | 0.196 |
| *TGFBRAP1* | rs17030766 | GG | 4.90±0.60(n=1874) | 6.03±1.35(n=607) | 9.78±3.15(n=203) | 8.88±3.17(n=97) |
|  |  | GA | 4.88±0.59(n=880) | 6.03±0.37(n=260) | 9.48±3.00(n=98) | 9.59±3.73(n=50) |
|  |  | AA | 4.91±0.47(n=98) | 6.15±0.40(n=30) | 10.99±3.16(n=18) | 5.77±0.76(n=2) |
|  |  | *F* | 0.288 | 1.703 | 2.382 | 1.214 |
|  |  | *P^a^* | 0.750 | 0.183 | 0.094 | 0.300 |
|  | rs2241797 | TT | 4.88±0.61(n=1510) | 6.05±0.37(n=438) | 10.02±3.30(n=1057) | 9.14±3.48(n=69) |
|  |  | TC | 4.90±0.59(n=1122) | 6.03±0.35(n=386) | 9.55±3.02(n=132) | 9.01±3.37(n=69) |
|  |  | CC | 5.01±0.45(n=220) | 5.99±0.33(n=73) | 9.49±3.10(n=28) | 9.09±2.98(n=11) |
|  |  | *F* | 5.098* | 0.396 | 0.508 | 0.046 |
|  |  | *P^a^* | 0.006 | 0.673 | 0.602 | 0.955 |
|  | rs2679860 | AA | 4.89±0.60(n=1953) | 6.04±0.37(n=602) | 9.74±3.23(n=202) | 9.16±3.38(n=96) |
|  |  | AG | 4.91±0.59(n=794) | 6.02±0.35(n=269) | 9.94±3.09(n=106) | 8.78±3.39(n=47) |
|  |  | GG | 4.99±0.46(n=102) | 6.11±0.35(n=27) | 8.52±2.35(n=11) | 10.05±3.43(n=6) |
|  |  | *F* | 1.910 | 0.730 | 0.644 | 0.170 |
|  |  | *P^a^* | 0.148 | 0.482 | 0.526 | 0.843 |

T2DM: type 2 diabetes mellitus; IFG: impaired fasting glucose; NGT: normal glucose tolerance; SNP, single nuclear polymorphisms;

WT: wild type; HT: heterozygote; MT: mutant type.

a. The comparisons of quantitative traits amongst genotypes of *TGFBR2* and *TGFBRAP1* after adjustment for age, gender, and BMI.

*. *P* <0.05 for trend test.

**Table S4. Comparisons of lginsulin amongst genotypes of *TGFBR2* and *TGFBRAP1***

| Gene | SNPs | Genotype | NGT | IFG | T2DM (non-treatment) | T2DM (treatment) |
| --- | --- | --- | --- | --- | --- | --- |
| *TGFBR2* | rs6785358 | AA | 0.66±0.31(n=1932) | 0.77±0.3(n=617) | 0.86±0.34(n=237) | 0.79±0.35(n=110) |
|  |  | AG | 0.67±0.32(n=635) | 0.77±0.31(n=207) | 0.84±0.34(n=67) | 0.90±0.34(n=34) |
|  |  | GG | 0.60±0.36(n=53) | 0.76±0.25(n=23) | 0.97±0.06(n=3) | 0.99(n=1) |
|  |  | *F* | 0.912 | 0.087 | 0.367 | 3.562 |
|  |  | *P^a^* | 0.402 | 0.917 | 0.693 | 0.031 |
|  | rs764522 | CC | 0.66±0.31(n=2023) | 0.77±0.30(n=644) | 0.86±0.34(n=248) | 0.79±0.33(n=115) |
|  |  | CG | 0.66±0.32(n=564) | 0.77±0.31(n=192) | 0.87±0.31(n=56) | 0.94±0.41(n=30) |
|  |  | GG | 0.66±0.36(n=33) | 0.74±0.25(n=11) | 0.90±0.04(n=3) | 1.01(n=1) |
|  |  | *F* | 0.002 | 0.052 | 0.084 | 2.154 |
|  |  | *P^a^* | 0.998 | 0.949 | 0.920 | 0.120 |
|  | rs9850060 | AA | 0.66±0.31(n=1716) | 0.76±0.3(n=524) | 0.87±0.33(n=200) | 0.84±0.36(n=87) |
|  |  | AG | 0.66±0.32(n=854) | 0.76±0.31(n=291) | 0.84±0.35(n=99) | 0.82±0.34(n=50) |
|  |  | GG | 0.63±0.33(n=131) | 0.80±0.31(n=34) | 0.76±0.26(n=11) | 0.63±0.34(n=9) |
|  |  | *F* | 0.461 | 0.457 | 0.751 | 2.073 |
|  |  | *P^a^* | 0.631 | 0.633 | 0.473 | 0.130 |
|  | rs3773645 | CC | 0.67±0.31(n=1237) | 0.75±0.31(n=386) | 0.86±0.32(n=135) | 0.83±0.33(n=74) |
|  |  | CG | 0.64±0.32(n=1183) | 0.79±0.30(n=373) | 0.88±0.34(n=145) | 0.84±0.38(n=55) |
|  |  | GG | 0.66±0.32(n=281) | 0.71±0.29(n=91) | 0.76±0.37(n=30) | 0.72±0.37(n=17) |
|  |  | *F* | 3.089 | 4.291 | 1.416 | 1.199 |
|  |  | *P^a^* | 0.046 | 0.014 | 0.244 | 0.304 |
|  | rs749794 | CC | 0.66±0.32(n=1223) | 0.77±0.30(n=363) | 0.83±0.29(n=126) | 0.83±0.40(n=59) |
|  |  | CT | 0.66±0.31(n=1179) | 0.77±0.30(n=404) | 0.86±0.36(n=146) | 0.77±0.31(n=71) |
|  |  | TT | 0.62±0.31(n=302) | 0.74±0.34(n=83) | 0.95±0.36(n=38) | 1.04±0.25(n=16) |
|  |  | *F* | 0.661 | 0.261 | 2.343 | 2.539 |
|  |  | *P^a^* | 0.516 | 0.770 | 0.098 | 0.083 |
|  | rs3773661 | GG | 0.66±0.31(n=1285) | 0.77±0.3(n=398) | 0.88±0.34(n=159) | 0.83±0.33(n=63) |
|  |  | GC | 0.66±0.31(n=1173) | 0.77±0.3(n=372) | 0.83±0.35(n=127) | 0.82±0.38(n=70) |
|  |  | CC | 0.63±0.34(n=246) | 0.73±0.32(n=80) | 0.90±0.27(n=24) | 0.80±0.35(n=13) |
|  |  | *F* | 1.374 | 0.416 | 1.901 | 0.190 |
|  |  | *P^a^* | 0.253 | 0.660 | 0.151 | 0.827 |
|  | rs11709624 | GG | 0.66±0.31(n=1316) | 0.76±0.30(n=417) | 0.86±0.33(n=166) | 0.86±0.37(n=65) |
|  |  | GC | 0.65±0.31(n=1143) | 0.77±0.31(n=369) | 0.86±0.34(n=121) | 0.82±0.35(n=62) |
|  |  | CC | 0.65±0.33(n=241) | 0.77±0.30(n=62) | 0.83±0.38(n=23) | 0.71±0.30(n=19) |
|  |  | *F* | 0.206 | 0.194 | 0.227 | 0.900 |
|  |  | *P^a^* | 0.824 | 0.824 | 0.797 | 0.409 |
|  | rs1155705 | GG | 0.65±0.32(n=1271) | 0.76±0.30(n=393) | 0.83±0.33(n=141) | 0.81±0.38(n=55) |
|  |  | GA | 0.67±0.31(n=1163) | 0.78±0.31(n=370) | 0.87±0.33(n=141) | 0.82±0.34(n=73) |
|  |  | AA | 0.63±0.33(n=269) | 0.73±0.32(n=86) | 0.92±0.36(n=28) | 0.9±0.35(n=18) |
|  |  | *F* | 1.554 | 0.064 | 1.048 | 0.389 |
|  |  | *P^a^* | 0.212 | 0.938 | 0.352 | 0.679 |
|  | rs1036096 | CC | 0.66±0.31(n=946) | 0.75±0.30(n=290) | 0.88±0.32(n=101) | 0.87±0.33(n=66) |
|  |  | CT | 0.64±0.32(n=1281) | 0.77±0.31(n=407) | 0.88±0.34(n=151) | 0.79±0.39(n=64) |
|  |  | TT | 0.67±0.30(n=477) | 0.78±0.30(n=153) | 0.76±0.34(n=58) | 0.78±0.28(n=16) |
|  |  | *F* | 2.114 | 0.023 | 1.679 | 0.658 |
|  |  | *P^a^* | 0.121 | 0.977 | 0.188 | 0.519 |
| *TGFBRAP1* | rs17030766 | GG | 0.66±0.32(n=1771) | 0.76±0.30(n=572) | 0.86±0.34(n=197) | 0.81±0.37(n=95) |
|  |  | GA | 0.64±0.31(n=838) | 0.78±0.31(n=251) | 0.82±0.31(n=95) | 0.86±0.29(n=49) |
|  |  | AA | 0.67±0.31(n=92) | 0.71±0.22(n=25) | 1.02±0.27(n=18) | 0.49±0.59(n=2) |
|  |  | *F* | 1.320 | 0.679 | 2.421 | 1.983 |
|  |  | *P^a^* | 0.267 | 0.507 | 0.091 | 0.142 |
|  | rs2241797 | TT | 0.661±0.32(n=1441) | 0.78±0.32(n=420) | 0.86±0.34(n=150) | 0.78±0.37(n=68) |
|  |  | TC | 0.65±0.31(n=1053) | 0.75±0.29(n=362) | 0.85±0.32(n=130) | 0.84±0.34(n=69) |
|  |  | CC | 0.63±0.33(n=208) | 0.74±0.22(n=67) | 0.84±0.38(n=28) | 0.94±0.31(n=9) |
|  |  | *F* | 0.670 | 0.548 | 0.030 | 0.450 |
|  |  | *P^a^* | 0.512 | 0.579 | 0.971 | 0.639 |
|  | rs2679860 | AA | 0.66±0.31(n=1861) | 0.77±0.30(n=568) | 0.86±0.32(n=196) | 0.81±0.36(n=95) |
|  |  | AG | 0.63±0.31(n=738) | 0.74±0.29(n=258) | 0.83±0.37(n=103) | 0.83±0.34(n=47) |
|  |  | GG | 0.65±0.33(n=99) | 0.77±0.27(n=23) | 0.91±0.19(n=11) | 0.88±0.26(n=4) |
|  |  | *F* | 1.351 | 0.433 | 0.226 | 0.052 |
|  |  | *P^a^* | 0.259 | 0.649 | 0.798 | 0.950 |

T2DM: type 2 diabetes mellitus; IFG: impaired fasting glucose; NGT: normal glucose tolerance; SNP, single nuclear polymorphisms;

WT: wild type; HT: heterozygote; MT: mutant type.

a. The comparisons of quantitative traits amongst genotypes of *TGFBR2* and *TGFBRAP1* after adjustment for age, gender, and BMI.

* *P* <0.05 for trend test.

**Table S5. Comparisons of lgHOMA-IR amongst genotypes of *TGFBR2* and *TGFBRAP1***

| Gene | SNPs | Genotype | NGT | IFG | T2DM (non-treatment) | T2DM (treatment) |
| --- | --- | --- | --- | --- | --- | --- |
| *TGFBR2* | rs6785358 | AA | -0.01±0.325(n=1932) | 0.19±0.30(n=617) | 0.48±0.37(n=237) | 0.37±0.37(n=110) |
|  |  | AG | 0.01±0.337(n=635) | 0.19±0.31(n=207) | 0.46±0.38(n=67) | 0.50±0.36(n=34) |
|  |  | GG | -0.06±0.388(n=53) | 0.19±0.25(n=23) | 0.59±0.12(n=3) | 0.38(n=1) |
|  |  | *F* | 0.714 | 0.120 | 0.244 | 4.286 |
|  |  | *P^a^* | 0.490 | 0.887 | 0.784 | 0.016 |
|  | rs764522 | CC | -0.01±0.33(n=2023) | 0.19±0.31(n=644) | 0.48±0.38(n=248) | 0.38±0.36(n=115) |
|  |  | CG | -0.01±0.33(n=564) | 0.20±0.31(n=192) | 0.48±0.34(n=56) | 0.50±0.41(n=30) |
|  |  | GG | 0.01±0.37(n=33) | 0.19±0.27(n=11) | 0.45±0.08(n=3) | 0.63(n=1) |
|  |  | *F* | 0.075 | 0.107 | 0.054 | 1.497 |
|  |  | *P^a^* | 0.928 | 0.898 | 0.947 | 0.227 |
|  | rs9850060 | AA | -0.01±0.33(n=1716) | 0.19±0.30(n=524) | 0.49±0.35(n=200) | 0.44±0.38(n=87) |
|  |  | AG | -0.01±0.34(n=854) | 0.19±0.31(n=291) | 0.47±0.42(n=99) | 0.38±0.37(n=50) |
|  |  | GG | -0.04±0.35(n=131) | 0.23±0.32(n=34) | 0.39±0.30(n=11) | 0.19±0.30(n=9) |
|  |  | *F* | 0.441 | 0.594 | 0.421 | 3.477* |
|  |  | *P^a^* | 0.643 | 0.552 | 0.657 | 0.034 |
|  | rs3773645 | CC | -0.01±0.33(n=1237) | 0.18±0.31(n=386) | 0.48±0.35(n=135) | 0.40±0.38(n=74) |
|  |  | CG | -0.03±0.34(n=1183) | 0.22±0.30(n=373) | 0.51±0.38(n=145) | 0.43±0.39(n=55) |
|  |  | GG | -0.01±0.34(n=281) | 0.14±0.3(n=91) | 0.36±0.38(n=30) | 0.35±0.33(n=17) |
|  |  | *F* | 2.545 | 4.014 | 1.256 | 0.873 |
|  |  | *P^a^* | 0.079 | 0.018 | 0.286 | 0.420 |
|  | rs749794 | CC | -0.01±0.34(n=1223) | 0.19±0.31(n=363) | 0.43±0.32(n=126) | 0.45±0.39(n=59) |
|  |  | CT | -0.01±0.33(n=1179) | 0.20±0.30(n=404) | 0.49±0.39(n=146) | 0.34±0.37(n=71) |
|  |  | TT | -0.05±0.33(n=302) | 0.17±0.34(n=83) | 0.57±0.43(n=38) | 0.55±0.27(n=16) |
|  |  | *F* | 0.632 | 0.226 | 3.328 | 1.446 |
|  |  | *P^a^* | 0.532 | 0.798 | 0.037 | 0.239 |
|  | rs3773661 | GG | -0.01±0.33(n=1285) | 0.20±0.31(n=398) | 0.50±0.38(n=159) | 0.40±0.33(n=63) |
|  |  | GC | -0.01±0.33(n=1173) | 0.19±0.30(n=372) | 0.45±0.37(n=127) | 0.41±0.42(n=70) |
|  |  | CC | -0.03±0.36(n=246) | 0.16±0.33(n=80) | 0.50±0.29(n=24) | 0.43±0.40(n=13) |
|  |  | *F* | 1.329 | 0.440 | 1.264 | 0.243 |
|  |  | *P^a^* | 0.265 | 0.644 | 0.284 | 0.785 |
|  | rs11709624 | GG | -0.01±0.33(n=1316) | 0.19±0.30(n=417) | 0.47±0.35(n=166) | 0.46±0.39(n=65) |
|  |  | GC | -0.01±0.33(n=1143) | 0.20±0.32(n=369) | 0.49±0.40(n=121) | 0.39±0.38(n=62) |
|  |  | CC | -0.02±0.35(n=241) | 0.19±0.31(n=62) | 0.47±0.40(n=23) | 0.26±0.27(n=19) |
|  |  | *F* | 0.133 | 0.175 | 0.152 | 1.921 |
|  |  | *P^a^* | 0.875 | 0.840 | 0.859 | 0.150 |
|  | rs1155705 | GG | -0.02±0.34(n=1271) | 0.19±0.30(n=393) | 0.44±0.35(n=141) | 0.41±0.38(n=55) |
|  |  | GA | -0.01±0.32(n=1163) | 0.20±0.31(n=370) | 0.49±0.37(n=141) | 0.4±0.38(n=73) |
|  |  | AA | -0.04±0.35(n=269) | 0.15±0.32(n=86) | 0.58±0.43(n=28) | 0.44±0.38(n=18) |
|  |  | *F* | 1.453 | 0.100 | 1.800 | 0.124 |
|  |  | *P^a^* | 0.234 | 0.905 | 0.167 | 0.883 |
|  | rs1036096 | CC | -0.01±0.33(n=946) | 0.18±0.30(n=290) | 0.50±0.36(n=101) | 0.45±0.41(n=66) |
|  |  | CT | -0.02±0.34(n=1281) | 0.20±0.31(n=407) | 0.50±0.39(n=151) | 0.35±0.37(n=64) |
|  |  | TT | -0.01±0.33(n=477) | 0.20±0.31(n=153) | 0.36±0.33(n=58) | 0.43±0.25(n=16) |
|  |  | *F* | 1.948 | 0.039 | 3.284 | 1.152 |
|  |  | *P^a^* | 0.143 | 0.961 | 0.039 | 0.319 |
| *TGFBRAP1* | rs17030766 | GG | -0.007±0.338(n=1775) | 0.188±0.307(n=578) | 0.48±0.382(n=181) | 0.385±0.376(n=101) |
|  |  | GA | -0.024±0.327(n=840) | 0.206±0.317(n=256) | 0.427±0.339(n=87) | 0.463±0.363(n=50) |
|  |  | AA | 0.005±0.316(n=92) | 0.159±0.224(n=25) | 0.694±0.358(n=18) | -0.095±0.54(n=2) |
|  |  | *F* | 1.502 | 0.438 | 3.843 | 3.407 |
|  |  | *P^a^* | 0.223 | 0.645 | 0.022 | 0.036 |
|  | rs2241797 | TT | -0.01±0.33(n=1441) | 0.20±0.32 (n=420) | 0.49±0.38(n=150) | 0.37±0.42(n=68) |
|  |  | TC | -0.02±0.33(n=1053) | 0.18±0.30(n=362) | 0.46±0.36(n=130) | 0.42±0.34(n=69) |
|  |  | CC | -0.02±0.34(n=208) | 0.17±0.23(n=67) | 0.45±0.39(n=28) | 0.55±0.34(n=9) |
|  |  | *F* | 0.176 | 0.572 | 0.018 | 0.575 |
|  |  | *P^a^* | 0.838 | 0.564 | 0.983 | 0.564 |
|  | rs2679860 | AA | -0.01±0.33(n=1861) | 0.20±0.31(n=568) | 0.48±0.36(n=196) | 0.40±0.40(n=95) |
|  |  | AG | -0.03±0.34(n=738) | 0.17±0.30(n=258) | 0.47±0.40(n=103) | 0.40±0.34(n=47) |
|  |  | GG | -0.01±0.35(n=99) | 0.21±0.28(n=23) | 0.48±0.23(n=11) | 0.56±0.23(n=4) |
|  |  | *F* | 1.159 | 0.461 | 0.151 | 0.332 |
|  |  | *P^a^* | 0.314 | 0.631 | 0.860 | 0.718 |

T2DM: type 2 diabetes mellitus; IFG: impaired fasting glucose; NGT: normal glucose tolerance; SNP, single nuclear polymorphisms;

WT: wild type; HT: heterozygote; MT: mutant type.

a. The comparisons of quantitative traits amongst genotypes of *TGFBR2* and *TGFBRAP1* after adjustment for age, gender, and BMI.

* *P* <0.05 for trend test.

**Table S6. Comparisons of lgHOMA-β amongst genotypes of *TGFBR2* and *TGFBRAP1***

| Gene | SNPs | Genotype | NGT | IFG | T2DM (non-treatment) | T2DM (treatment) |
| --- | --- | --- | --- | --- | --- | --- |
| *TGFBR2* | rs6785358 | AA | 1.83±0.34(n=1881) | 1.67±0.3(n=617) | 1.42±0.38(n=237) | 1.44±0.45(n=110) |
|  |  | AG | 1.83±0.34(n=619) | 1.67±0.31(n=207) | 1.39±0.37(n=67) | 1.50±0.45(n=34) |
|  |  | GG | 1.72±0.36(n=51) | 1.65±0.27(n=23) | 1.50±0.04(n=3) | 2.00(n=1) |
|  |  | *F* | 2.398 | 0.044 | 0.405 | 1.785 |
|  |  | *P^a^* | 0.091 | 0.957 | 0.668 | 0.172 |
|  | rs764522 | CC | 1.83±0.34(n=1967) | 1.67±0.30(n=644) | 1.41±0.39(n=248) | 1.41±0.42(n=115) |
|  |  | CG | 1.81±0.34(n=551) | 1.67±0.31(n=192) | 1.44±0.36(n=56) | 1.60±0.53(n=30) |
|  |  | GG | 1.8±0.36(n=33) | 1.60±0.23(n=11) | 1.55±0.03(n=3) | 1.54(n=1) |
|  |  | *F* | 0.468 | 0.095 | 0.354 | 1.721 |
|  |  | *P^a^* | 0.627 | 0.910 | 0.702 | 0.183 |
|  | rs9850060 | AA | 1.82±0.34(n=1670) | 1.66±0.30(n=524) | 1.44±0.39(n=200) | 1.44±0.45(n=87) |
|  |  | AG | 1.82±0.34(n=833) | 1.67±0.31(n=291) | 1.38±0.36(n=99) | 1.51±0.45(n=50) |
|  |  | GG | 1.81±0.35(n=129) | 1.69±0.3(n=34) | 1.29±0.26(n=11) | 1.26±0.45(n=9) |
|  |  | *F* | 0.379 | 0.231 | 0.707 | 0.587 |
|  |  | *P^a^* | 0.685 | 0.793 | 0.494 | 0.557 |
|  | rs3773645 | CC | 1.83±0.34(n=1200) | 1.65±0.30(n=386) | 1.42±0.39(n=135) | 1.50±0.43(n=74) |
|  |  | CG | 1.81±0.35(n=1156) | 1.69±0.30(n=373) | 1.42±0.36(n=145) | 1.46±0.46(n=55) |
|  |  | GG | 1.83±0.34(n=276) | 1.61±0.29(n=91) | 1.35±0.43(n=30) | 1.26±0.49(n=17) |
|  |  | *F* | 2.282 | 4.732 | 1.184 | 2.133 |
|  |  | *P^a^* | 0.102 | 0.009 | 0.307 | 0.122 |
|  | rs749794 | CC | 1.82±0.34(n=1192) | 1.67±0.3(n=363) | 1.40±0.34(n=126) | 1.39±0.51(n=59) |
|  |  | CT | 1.83±0.34(n=1151) | 1.67±0.3(n=404) | 1.40±0.42(n=146) | 1.43±0.38(n=71) |
|  |  | TT | 1.79±0.36(n=292) | 1.63±0.34(n=83) | 1.49±0.32(n=38) | 1.80±0.39(n=16) |
|  |  | *F* | 0.668 | 0.368 | 0.606 | 5.412* |
|  |  | *P^a^* | 0.513 | 0.692 | 0.546 | 0.005 |
|  | rs3773661 | GG | 1.82±0.34(n=1255) | 1.67±0.30(n=398) | 1.43±0.37(n=159) | 1.49±0.44(n=63) |
|  |  | GC | 1.83±0.35(n=1141) | 1.66±0.30(n=372) | 1.37±0.38(n=127) | 1.44±0.47(n=70) |
|  |  | CC | 1.80±0.35(n=239) | 1.64±0.32(n=80) | 1.5±0.41(n=24) | 1.36±0.41(n=13) |
|  |  | *F* | 1.704 | 0.348 | 2.076 | 0.249 |
|  |  | *P^a^* | 0.182 | 0.706 | 0.127 | 0.780 |
|  | rs11709624 | GG | 1.82±0.35(n=1283) | 1.66±0.29(n=417) | 1.43±0.38(n=166) | 1.45±0.44(n=65) |
|  |  | GC | 1.82±0.34(n=1112) | 1.67±0.31(n=369) | 1.41±0.37(n=121) | 1.49±0.47(n=62) |
|  |  | CC | 1.82±0.34(n=236) | 1.67±0.30(n=62) | 1.35±0.42(n=23) | 1.38±0.46(n=19) |
|  |  | *F* | 0.673 | 0.236 | 0.392 | 0.289 |
|  |  | *P^a^* | 0.511 | 0.790 | 0.676 | 0.749 |
|  | rs1155705 | GG | 1.81±0.34(n=1236) | 1.66±0.3(n=393) | 1.39±0.39(n=141) | 1.41±0.51(n=55) |
|  |  | GA | 1.83±0.35(n=1136) | 1.68±0.3(n=370) | 1.43±0.38(n=141) | 1.45±0.41(n=73) |
|  |  | AA | 1.8±0.34(n=262) | 1.64±0.32(n=86) | 1.41±0.33(n=28) | 1.6±0.42(n=18) |
|  |  | *F* | 2.062 | 0.015 | 0.315 | 0.963 |
|  |  | *P^a^* | 0.127 | 0.985 | 0.730 | 0.384 |
|  | rs1036096 | CC | 1.82±0.34(n=922) | 1.65±0.29(n=290) | 1.44±0.38(n=101) | 1.50±0.36(n=66) |
|  |  | CT | 1.81±0.35(n=1250) | 1.67±0.31(n=407) | 1.42±0.36(n=151) | 1.45±0.53(n=64) |
|  |  | TT | 1.84±0.33(n=463) | 1.68±0.3(n=153) | 1.34±0.41(n=58) | 1.28±0.41(n=16) |
|  |  | *F* | 1.102 | 0.040 | 0.150 | 2.400 |
|  |  | *P^a^* | 0.332 | 0.961 | 0.861 | 0.094 |
| *TGFBRAP1* | rs17030766 | GG | 1.82±0.34(n=1725) | 1.66±0.30(n=572) | 1.40±0.38(n=197) | 1.44±0.47(n=95) |
|  |  | GA | 1.81±0.33(n=816) | 1.67±0.30(n=251) | 1.40±0.39(n=95) | 1.47±0.41(n=49) |
|  |  | AA | 1.84±0.36(n=91) | 1.58±0.23(n=25) | 1.50±0.27(n=18) | 1.45±0.74(n=2) |
|  |  | *F* | 0.450 | 1.498 | 0.149 | 0.280 |
|  |  | *P^a^* | 0.638 | 0.224 | 0.862 | 0.757 |
|  | rs2241797 | TT | 1.83±0.35(n=1403) | 1.67±0.31(n=420) | 1.40±0.38(n=150) | 1.42±0.44(n=68) |
|  |  | TC | 1.81±0.32(n=1025) | 1.65±0.29(n=362) | 1.41±0.34(n=130) | 1.47±0.46(n=69) |
|  |  | CC | 1.77±0.35(n=205) | 1.65±0.22(n=67) | 1.42±0.47(n=28) | 1.52±0.37(n=9) |
|  |  | *F* | 3.188* | 0.488 | 0.120 | 0.051 |
|  |  | *P^a^* | 0.041 | 0.614 | 0.887 | 0.950 |
|  | rs2679860 | AA | 1.83±0.34(n=1814) | 1.67±0.30(n=568) | 1.42±0.36(n=196) | 1.44±0.45(n=95) |
|  |  | AG | 1.79±0.33(n=716) | 1.65±0.29(n=258) | 1.36±0.40(n=103) | 1.48±0.45(n=47) |
|  |  | GG | 1.81±0.34(n=99) | 1.66±0.28(n=23) | 1.57±0.34(n=11) | 1.32±0.36(n=4) |
|  |  | *F* | 2.258 | 0.417 | 0.808 | 0.277 |
|  |  | *P^a^* | 0.105 | 0.659 | 0.447 | 0.759 |

T2DM: type 2 diabetes mellitus; IFG: impaired fasting glucose; NGT: normal glucose tolerance; SNP, single nuclear polymorphisms;

WT: wild type; HT: heterozygote; MT: mutant type.

a. The comparisons of quantitative traits amongst genotypes of *TGFBR2* and *TGFBRAP1* after adjustment for age, gender, and BMI.

* *P* <0.05 for trend test.

**Table S7. miRNA profiles comparison between T2DM and NGT.**

| miRNA | T2DM | NGT | *t* | *P* |
| --- | --- | --- | --- | --- |
| has-miR-150-5p* | 10.75±2.27 | 8.73±2.47 | 5.165 | <0.001 |
| has-miR-328-3p | 10.11±2.45 | 7.98±2.24 | 5.541 | <0.001 |
| has-miR-335-5p | 10.68±2.33 | 9.88±2.19 | 2.093 | 0.038 |
| has-miR-30b-5p* | 75.07±19.25 | 48.50±22.53 | 7.720 | <0.001 |
| has-miR-93-5p* | 23.48±6.47 | 18.97±6.16 | 4.316 | <0.001 |
| has-miR-511-5p* | 6.47±2.00 | 7.19±1.70 | 2.348 | 0.020 |
| has-miR-126-3p* | 16.69±5.18 | 18.70±4.90 | 2.405 | 0.017 |
| has-miR-320a* | 8.24±2.89 | 6.52±2.86 | 3.619 | <0.001 |
| has-let-7g-5p* | 22.71±8.63 | 20.84±8.12 | 1.330 | 0.186 |
| has-miR-139-5p* | 63.58±13.67 | 48.12±15.92 | 6.200 | <0.001 |
| has-miR-191-5p | 11.16±1.78 | 8.95±2.05 | 6.895 | <0.001 |
| has-miR-574-3p | 12.79±1.97 | 10.84±2.29 | 5.540 | <0.001 |
| has-miR-628-3p | 12.69±2.63 | 13.50±3.78 | 1.202 | 0.232 |
| has-miR-1274B | 2.66±1.53 | 3.07±1.97 | 1.404 | 0.163 |
| has-miR-720 | 6.86±1.50 | 5.64±1.64 | 4.470 | <0.001 |

A total of 10 miRNAs in T2DM showed significantly higher expression levels than controls, whereas 2 miRNAs expression were significantly lower than controls. * refers to the skewed distribution of miRNA expression, which was transformed by the Box-cox model into normal distribution. T2DM: type 2 diabetes mellitus; NGT: normal glucose tolerance.

**Table S8. Comparisons of miRNA expression amongst genotypes of *TGFBR2* rs749794 and *TGFBRAP1* rs2241797**

| miRNA | SNPs | Genotype | T2DM | NGT |
| --- | --- | --- | --- | --- |
| has-miR-30b-5p | rs749794 | CC | 82.24±20.30 (n=28) | 48.26±21.38 (n=43) |
|  | (*TGFBR2*) | CT | 71.12±14.04 (n=40) | 48.21±19.84 (n=20) |
|  |  | TT | 69.00±32.35 (n=7) | 50.11±33.10 (n=10) |
|  |  | *F* | 3.332 | 0.029 |
|  |  | *P* | 0.041 | 0.971 |
| has-miR-139-5p |  | CC | 68.14±16.05 (n=41) | 43.79±14.90 (n=26) |
|  |  | CT | 62.59±13.68 (n=22) | 52.67±15.63 (n=35) |
|  |  | TT | 63.58±13.67 (n=12) | 54.56±16.57 (n=7) |
|  |  | *F* | 2.525 | 6.566* |
|  |  | *P* | 0.088 | 0.012 |
| has-miR-720 |  | CC | 7.26±1.70 (n=28) | 5.30±2.09 (n=30) |
|  |  | CT | 6.72±1.32 (n=40) | 6.12±0.92 (n=18) |
|  |  | TT | 6.05±1.31 (n=7) | 5.78±0.75 (n=10) |
|  |  | *F* | 4.467* | 1.480 |
|  |  | *P* | 0.038 | 0.237 |
| has-miR-30b-5p | rs2241797 | TT | 71.88±21.66 (n=42) | 44.18±21.51 (n=37) |
|  | (*TGFBRAP1*) | TC | 80.30±14.53 (n=29) | 49.22±18.97 (n=27) |
|  |  | CC | 70.69±18.00 (n=4) | 64.09±30.93 (n=9) |
|  |  | *F* | 1.786 | 5.310* |
|  |  | *P* | 0.175 | 0.024 |
| has-miR-93-5p |  | TT | 22.13±6.32 (n=40) | 17.31±6.31 (n=37) |
|  |  | TC | 25.40±6.73 (n=29) | 20.24±5.40 (n=27) |
|  |  | CC | 23.02±1.06 (n=4) | 21.95±6.26 (n=9) |
|  |  | *F* | 2.231 | 6.141* |
|  |  | *P* | 0.115 | 0.016 |

T2DM: type 2 diabetes mellitus; IFG: impaired fasting glucose; NGT: normal glucose tolerance; SNP, single nuclear polymorphisms.

*. *P* <0.05 for trend test.

**Figure S1**. Regional LD Plot for the three positive SNPs rs749794, rs2241797 and rs2679860
